# The use of free buccal pad fat graft as a viable therapeutic modality in localized gingival recession: a randomized controlled clinical trial

**DOI:** 10.1186/s12903-025-06150-8

**Published:** 2025-05-24

**Authors:** Reham Abdel-Fatah, Ayman Elkashty, Hesham El-Sharkawy

**Affiliations:** Oral Medicine, Periodontology, Diagnosis, and Oral Radiology Depatment, Faculty of Dentistry, Mansoura University, Algomhoria St, Mansoura City, Aldakhlia 35516 Egypt

**Keywords:** Free buccal pad fat graft, Coronally advanced flap, De-epithelialized free gingival graft, Localized gingival recession

## Abstract

**Background:**

The buccal fat pad (BFP) is a unique, encapsulated fatty tissue that retains its volume and structure over time and contains undifferentiated mesenchymal cells. It has been used in various surgical techniques, such as closing oroantral and oronasal communications and repairing defects due to trauma or malignancy. A few years ago, the buccal fat pad was utilized as a pedicle or free soft tissue graft to treat gingival recessions. Therefore, this randomized clinical trial (RCT) was conducted to assess the effectiveness of the free buccal fat pad graft (FBPFG) compared to the de-epithelialized free gingival graft (DFGG) in conjunction with coronally advanced flap (CAF) in localized gingival recession treatment.

**Materials and methods:**

This RCT included 39 participants, with 20 patients receiving a coronally advanced flap (CAF) with FBPFG (Group I) and 19 patients treated with CAF and DFGG (Group II). Clinical parameters such as probing depth (PD), clinical attachment level (CAL), recession depth (RD), width of keratinized gingiva (WKG), and gingival thickness (GT) were assessed after 3- and 6 months of surgical intervention.

**Results:**

After 3- and 6-month follow-ups, statistically significant changes were noted between the groups in all clinical parameters. At 1st and 2nd days of surgical intervention, there was a statistically significant decrease in the values of the visual analogue scale (VAS) in group I compared to group II. Regarding dentinal hypersensitivity, there were statistically significant variations of 3- and 6-month parameters compared to baseline in the two groups. However, there were no differences between the two after the therapy.

**Conclusion:**

It was proven that DFGG produces significantly better outcomes than FBPFG in treating localized gingival recession. However, FBPFG might serve as a viable alternative for mild to moderate gingival recession cases, where only a limited reduction in recession depth and modest increase in gingival thickness and width of keratinized tissues are required, with the benefit of lower donor site morbidity.

**Trial registration:**

This randomized controlled clinical trial was registered on the Pan African Clinical Trials Registry and approved on 22/06/2023 (Trial no: PACTR202307891237031).

**Supplementary Information:**

The online version contains supplementary material available at 10.1186/s12903-025-06150-8.

## Introduction

Gingival recession (GR) refers to the marginal gingival tissue apical displacement below the cementoenamel junction (CEJ), causing root surface exposure. Hence, various sequelae might occur, such as esthetic concerns, root caries, dentin hypersensitivity, and challenges in maintaining proper oral hygiene. Moreover, severe pulpal exposure may result in significant pain [1, 2]. GR is a prevalent condition all over the world caused by several etiological factors, including faulty tooth brushing, plaque-induced inflammation, root prominence due to orthodontic tooth movement, bone dehiscence, frenal pull, and high muscle attachment, defective restorations extending subgingivally, and eventually destructive periodontal disease [3–6].

Since 1968, GR has been classified by various investigators, beginning with Sullivan and Atkins, until Miller's classification (1985), the most widely accepted classification for over two decades [7]. Nevertheless, Miller’s classification has gained popularity among clinicians, it has some evident drawbacks, such as reliance on MGJ as a reference, which might be challenging to identify[8]. Hence, Cairo et al. [9] proposed a new GR classification based on interproximal and buccal attachment loss, which relies on a treatment-oriented background to overcome the previous limitations. It was categorized into three classes: RT1 (no attachment loss interproximally), RT2 (equal interproximal loss to or less than buccal loss), and RT3 (interproximal loss greater than loss buccally) [9].

Various treatment modalities for GR exist, including non-surgical and surgical options, with the coronally advanced flap (CAF) as one of the most recognized surgical techniques. CAF is often used alone or in conjunction with different biomaterials, such as the gold standard autologous connective tissue graft (CTG), which can be harvested as solely sub-epithelial CTG or de-epithelialized free gingival graft (DFGG) [10]. Sub-epithelial CTG harvesting is encouraged in cases of adequate palatal fibromucosa thickness to allow for sufficient tissue grafting and remaining palatal soft tissue coverage.

Although, in thin palatal tissues, it is impossible to harvest adequate soft tissue thickness without palatal necrosis and post-operative pain, so DFGG is used as an alternative for CTG, which allows for harvesting free gingival graft and then de-epithelialized with the blade [11]. Additionally, DFGG involves the superficial denser, firmer, and more stable connective tissue closer to epithelium with better quality and promising root coverage results [11, 12]. A recent systematic review has proposed a buccal fat pad graft as an alternative to CTG in GR treatment to overcome the shortcomings of pain, discomfort, and patient morbidity following palatal tissue harvesting [13].

The buccal fat pad (BFP) is a specialized adipose tissue on both sides of the buccal space. It is notable for its lipid content, which is not influenced by regular fat metabolism (lipolysis), and its role in facilitating intermuscular movement [14–16]. Most importantly, it has been shown that the BFP contains undifferentiated mesenchymal stem cells, which have the potential for differentiation and regeneration, making it a promising site for adipose grafting and osteocartilage regeneration [17]. Furthermore, adipocytes from the BFP exhibit increased osteoblastic activity, indicating their potential in regenerative applications [18].

Since its initial use by Egyedi for closing oro-antral communications, the BFP has been utilized in various surgical procedures, particularly for repairing defects from malignant or traumatic lesions [19]. Few previous studies have explored applying BFP as pedicled or non-pedicled grafts in treating different Miller’s classes of gingival recession [20–24]. Therefore, this study hypothesized that the free BFP graft (FBPFG) might serve as a viable and less invasive surgical treatment modality for GR when used in combination with coronally advanced flap (CAF), compared to the use of de-epithelialized free gingival graft (DFGG) harvested from the palate.

## Materials and methods

### Study populations

This randomized controlled clinical trial was registered on the Pan African Clinical Trials Registry and approved on 17/07/2023 (Trial no: PACTR202307891237031). This trial included forty patients with localized gingival recession defects carefully chosen from the Department of Oral Medicine and Periodontology outpatient clinic, Faculty of Dentistry, Mansoura University, from 1 August 2023 to 30 April 2024. The subjects were informed about the details of the treatment protocol they underwent, including the procedures carried out, potential risks, and alternative treatment options. They could provide written informed consent before any necessary procedure was conducted. They agreed to attend the follow-up visits as mandated by the Faculty of Dentistry ethical committee of Mansoura University (Ethical committee approval number: M0306023OM).

The inclusion criteria involved localized Cairo recession defect RT1 ≥ 2 mm in a tooth with a probing depth (PD) of < 3 mm, the presence of at least 1 mm of keratinized tissue located above the root exposure [25], no cervical region restorations, and bleeding on probing. All participants were between (18–45 years old), and systemically healthy. Additionally, the participants were periodontal healthy individuals with no periodontal surgery contraindications. They had not used any medications that might affect the condition of their gingival or periodontal tissues and had not experienced any previous periodontal surgery in the areas of interest.

The exclusion criteria included multiple adjacent gingival recessions, lingual recession in the selected teeth, systemic chronic conditions linked to periodontitis or systemic inflammation, presence of malocclusion/ongoing orthodontic therapy, previous history of periodontal disease/surgical periodontal therapy, smoking status, and pregnancy.

### Randomization and allocation concealment

Selected patients were randomly assigned to treatment with coronally advanced flap in conjunction with either FBFPG or DFGG by a central registrar of the Oral Medicine and Periodontology department using a computer-generated randomization sequence. This sequence was kept in opaque, closed envelopes containing the patient numbers and randomization treatment codes. The treatment code was disclosed only immediately before surgery, as the treatment allocation was masked from the surgeon (R.A.) until the envelopes were opened after the initial stages of the surgical procedure (flap advancement and root conditioning). Nevertheless, the investigators (A.E.) and (H.E.) responsible for patient recruitment and clinical measurements were blinded to the treatment until the statistical analysis was completed.

### Study design

All patients underwent a comprehensive periodontal examination during the inclusion screening phase. Once the selected patients consented to participate in the study, they received personalized oral hygiene instructions, which included guidance on proper brushing techniques and dental prophylaxis with polishing. The selected individuals were allocated into two groups; Group I consisted of twenty subjects who received treatment with a coronally advanced flap and free buccal pad fat graft, whereas Group II included nineteen participants who were treated with a coronally advanced flap and de-epithelialized free gingival graft.

### Clinical assessment

All measurements were recorded pre-surgically (after phase I therapy) and postoperatively at three and six months with a calibrated UNC—15 probe (Hu-Friedy, Chicago, USA) except gingival thickness, visual analog scale, and dentinal hypersensitivity.

1. Recession Depth (RD) measured from the cementoenamel junction (CEJ) to the most apical extension of the gingival margin at the mid-buccal site. The calculated reduction of recession depth after 6 months represents the clinical trial's primary outcome.
2. Probing Depth (PD) measured from the free margin of the gingiva to the sulcus base at the mid-buccal site.
3. Clinical Attachment Level (CAL) measured from the cementoenamel junction to the base of the sulcus (RD + PD).
4. Width of keratinized gingiva (WKG); measured from the free gingival margin to the mucogingival junction at the mid-buccal point.
5. Gingival thickness (GT) was measured under local anesthesia using an endodontic spreader (#25) with a stopper 2 mm from the buccal gingival margin. Then, the distance between the stopper and the spreader tip (GT) was measured with a digital clipper (Bacolis, China).
6. Visual analog scale (VAS) scale [26]; to assess post-operative pain, patients were asked about their pain levels, swelling, and overall experiences related to the surgical technique and instruments used. The discomfort was measured using a Visual Analog Scale (VAS), which ranged from “no discomfort” (score 0) to “unbearable discomfort” (score 10). Patients completed the questionnaires following the surgery on the 1st, 2nd, 4th, 7th, and 14th days.
7. Dentinal hypersensitivity (DH) score; discomfort levels were recorded after a dental syringe directed a blast of air toward the root surface for one second. The syringe was positioned at a 90° angle, 2–3 mm from the root. Neighboring teeth were protected during the procedure by the dentist's gloved fingers. Following this, the patient was asked to rate their discomfort. The perceived discomfort was evaluated through a Visual Analog Scale (VAS), marked with “no pain” at one end (score 0) and “unbearable pain” at the other end (score 10).

RD reduction is the primary outcome in this RCT, whereas the secondary outcomes include PD reduction, CAL gain, DH decrease and WKG and GT increase.

### Surgical procedures

#### A) Coronally advanced flap (CAF) procedure

The trapezoidal CAF procedure stated by Zucchelli and his colleagues [27] was used in all surgical procedures in both groups. The design of the flap was a split-full-split thickness incision flap as the surgical papillae were reflected as split thickness using a 15-c blade (Kiato, Kehr Surgical Private Limited, India) by tracing a 3 mm horizontal incision at the base of each anatomical papillae at a distance equal to recession depth plus 1 mm from the tip of the papillae. Two vertical, slightly divergent releasing incisions at the end of both horizontal incisions were completed until the alveolar mucosa.

The soft tissues located apical to root exposure were elevated to full thickness to expose the alveolar bone crest. Then, the facial portion of the interdental papillae was de-epithelialized to create a connective tissue bed for CAF suturing. As well as the exposed root surface was cautiously instrumented with #5/6 Gracy curette (Zeffiro, LASCOD, Florence, Italy) and then conditioned with ethylene diamine tetraacetic acid (EDTA, METABIOMED CO., LTD, Korea) to eliminate the smear layer, and thoroughly flushed with sterile saline [27].

**Fig. 1:**
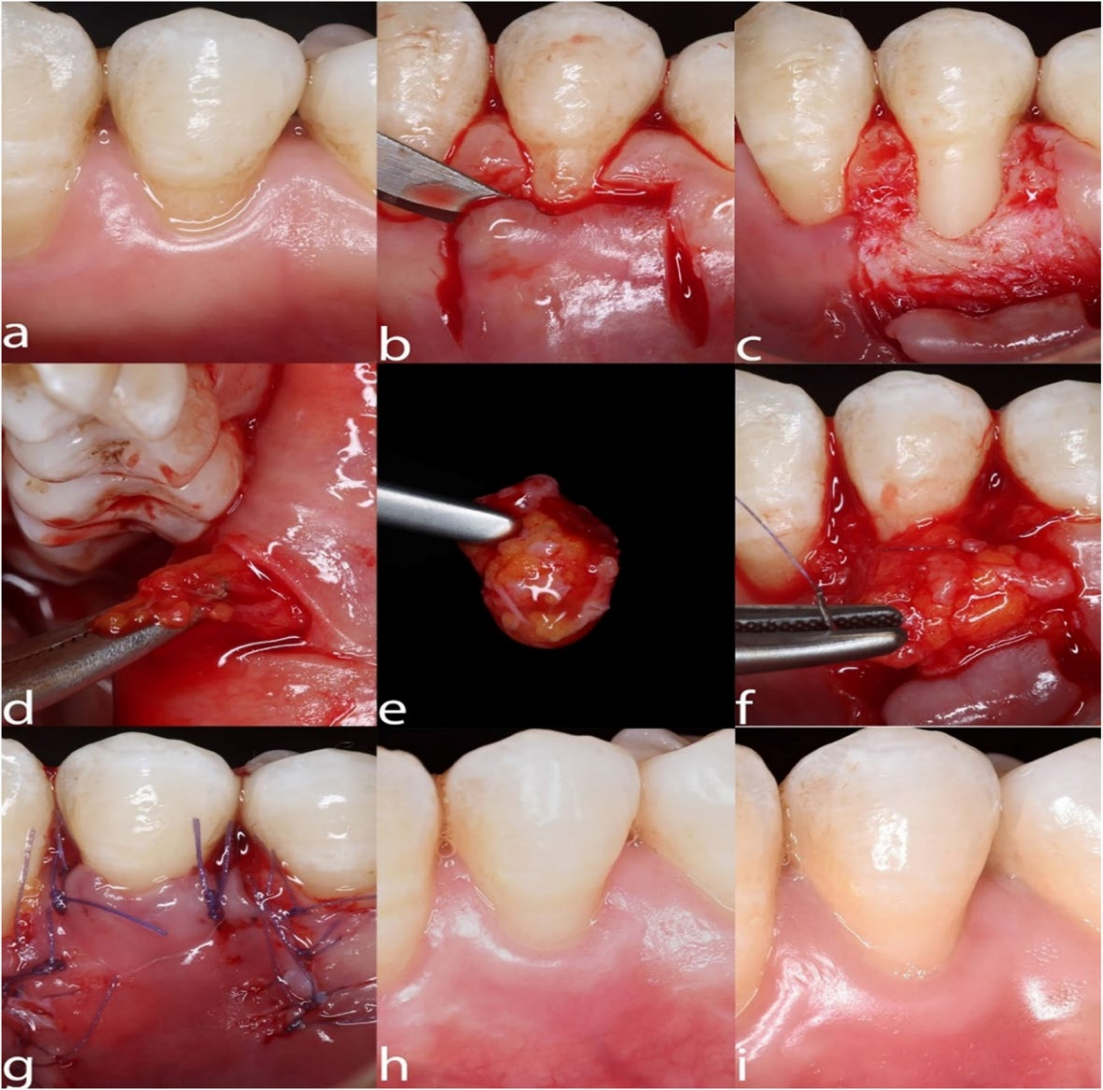
FBPFG case showing **a**) baseline, **b**) CAF incision, **c**) split-thickness flap reflection, **d**) FBPFG harvesting, **e**) FBPFG, **f**) FBPFG suturing, **g**) flap suturing, **h**) 3-month follow-up, **i**) 6-month follow-up

**Fig. 2:**
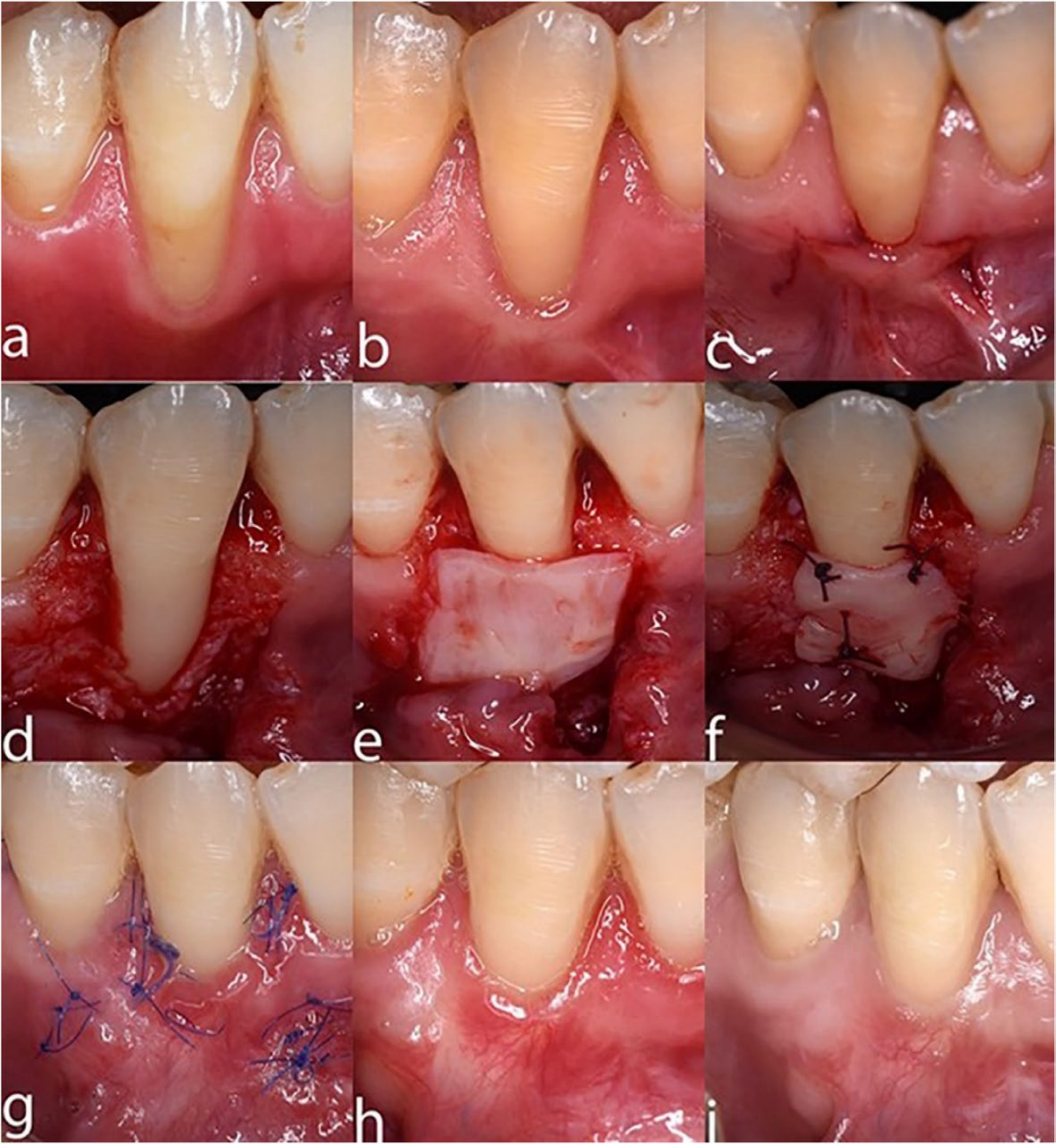
DFGG case showing **a**, **b**) baseline, **c**) CAF incision, **d**) split-thickness flap reflection, **e**) DFGG harvested, **f**) DFGG suturing, **g**) 2 weeks post-operative, **h**) 3-month follow-up, **i**) 6-month follow-up

After harvesting the graft either FBPFG or DFGG and suturing it to cover the exposed area of the root surface with an absorbable 6–0 suture (EGYSORB, Braided Polyglycolic acid, Egypt), the overlying flap was then secured to the interdental papilla bed using a non-absorbable 6–0 suture (EGYPROLENE, synthetic monofilament polypropylene, Egypt) (as shown in Figs. 1 and 2).

#### B) Free buccal pad fat graft (FBPFG)

Anesthetic infiltration was administered at the designated donor site on the cheek mucosa. Following this, a 1.5 cm horizontal incision was made below the opening of Stenson's duct using a 15c blade. A curved hemostat was employed to temporarily reposition the intervening muscles, allowing for exposure of the adipose tissue. A 1.5 × 1.5 cm section of adipose tissue was then excised using scissors (Goldman Fox, MEDSEY, Maniago, Italy). The area was pressed to facilitate the wound margins closure, and the adjacent tissues were sutured together using 6–0 non-absorbable sutures [28].

#### C) De-epithelialized free gingival graft (DFGG) harvesting

To harvest the free gingival graft (FGG), a surgical site was prepared secondarily on the palatal mucosa following the technique defined by Zucchelli et al. Two vertical and two horizontal incisions were made to outline the graft, with a horizontal incision coronally. The 15c blade was held perpendicular to the palatal surface until sufficient soft tissue thickness was achieved, after which it was rotated to be nearly parallel to the superficial tissues.

Once harvested, the FGG was de-epithelialized with a 15c blade, and any underlying adipose/glandular tissues were removed to obtain DFGG with a uniform thickness measured approximately (1.5–2 mm). The palatal wound was then secured with gel foam (Surgispon ®, Aegis Lifesciences, Gujarat, India) and dressing sutures [11].

### Post-operative protocol

All patients received post-operative instructions that included applying an ice pack to the surgical site for the first four hours, following a liquid and/or soft food diet for three days, avoiding teeth brushing in the surgical area, and rinsing with a 0.12% chlorhexidine digluconate mouthwash for one minute every 12 h for two weeks. They were also prescribed post-operative medications, including amoxicillin 500 mg every eight hours for seven days or clindamycin 300 mg twice daily for seven days for those who were allergic to penicillin, as well as ibuprofen 600 mg every eight hours for three days. Sutures were scheduled for removal 14 days after surgery, and patients were advised to brush gently their teeth with a soft-bristled toothbrush. Furthermore, all patients were informed about a monthly program for professional tooth cleaning and oral hygiene instructions scheduled to occur from the first month through the sixth month after surgery.

### Statistical analysis

Sample size calculation was based on the root coverage percentage post-operatively retrieved from previous research (Bin et al., 2023). Using G power program version 3.1.9.4 for sample size calculation based on the effect size of 0.97(50 ± 36.01 & 81.3 ± 27.53), using the 2-tailed test, α error = 0.05 and power = 80.0%, the total calculated sample size was 18 in each group [29]. To compensate for future dropouts, twenty-one cases were treated in each group. Data were provided to the computer and explored using IBM SPSS software package version 20.0 (Armonk, NY: IBM Corp). Qualitative data were illustrated using numbers and percentages. Shapiro–Wilk test was used to verify the normality of distribution. Quantitative data were designated using mean and standard deviation. The Chi-square, Fisher’s Exact, Student t-test, and Mann- Whitney U tests were accomplished for suitable data sets. At the 5% level of significance, the outcome results were judged.

## Results

### Study population

**Fig. 3:**
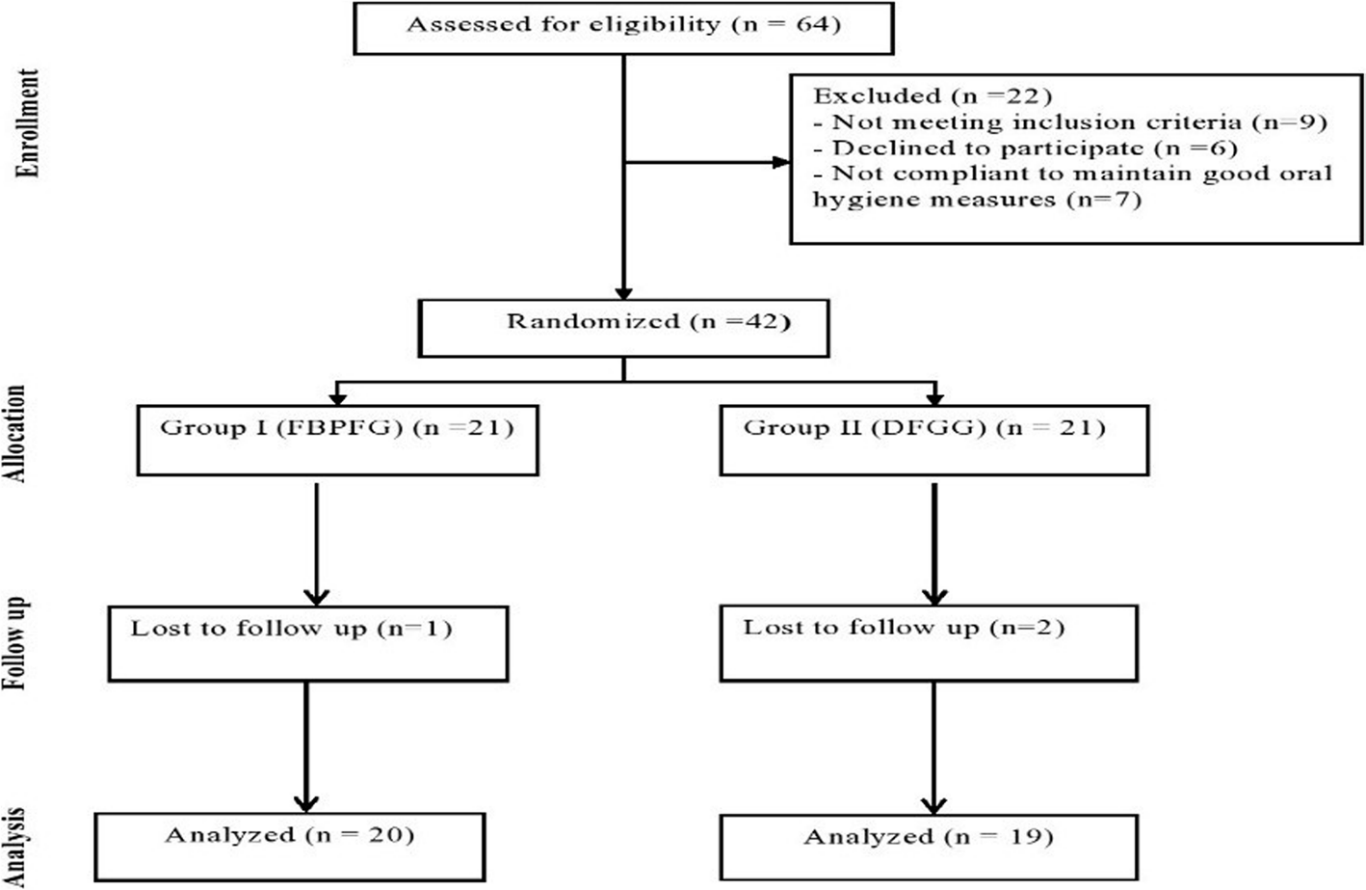
RCT Flowchart

Sixty-four participants were initially chosen from the periodontology outpatient clinic at the Faculty of Dentistry, Mansoura University, and assessed for eligibility to be enrolled in the trial. After the screening phase, twenty-two participants were omitted; nine did not meet the inclusion criteria, six refused to participate, and seven could not maintain proper oral hygiene. The randomization process involved forty-two participants evenly distributed between two study groups, with twenty-one participants in each group. During the follow-up period, one participant from the FBPFG group dropped out due to travelling abroad. In contrast, in the DFGG group, two individuals were lost as one of them had significant body fractures due to a car accident, and the other one declined to complete the study for no apparent reason. Hence, thirty-nine participants, twenty in the FBPFG group and nineteen in the DFGG group, were analyzed (as shown in Fig. 3).

### Demographic data and baseline characteristics

Demographic and baseline periodontal features for enrolled participants in the present clinical trial are exhibited in Table 1. Group I (FBPFG) and Group II (DFGG) showed no significant statistical variations regarding gender and age. Furthermore, Table 1 shows the mean values (± SD) of probing depth (PD), clinical attachment level (CAL), recession depth (RD), gingival thickness (GT), and width of keratinized gingiva (WKG) of the study groups. All previously mentioned measurements in group I revealed no statistically significant changes compared to the group II population, *p* > 0.05.

**Table 1:** Demographic data of the study population and baseline periodontal features (M ± SD)

Variable	Group I (FBPFG) (*n* = 20)	Group II (DFGG) (*n* = 19)	*p*
Gender			
* Male/Female (n)*	9/11	7/12	*NS*
Age (years)	30.30 ± 9.36	35.70 ± 10.94	*NS*
Age range (years)	24–43	27- 49	*N/A*
PD (mm)	1.86 ± 0.58	1.93 ± 0.63	*NS*
CAL (mm)	4.70 ± 0.95	4.90 ± 0.74	*NS*
RD (mm)	3.54 ± 0.74	3.30 ± 0.69	*NS*
GT (mm)	1.0 ± 0.21	1.05 ± 0.16	*NS*
WKG (mm)	2.70 ± 0.69	2.50 ± 0.58	*NS*

*PD* probing depth, *CAL* clinical attachment level, *RD* Recession depth, *GT* gingival thickness, *WKG* width of keratinized gingiva

*NS* non-significant difference at *P* > 0.05 (there was an insignificant difference between both groups at baseline regarding all measured parameters)

N/A: not applicable (in terms of age range)

### Clinical trial outcomes

**Fig. 4:**
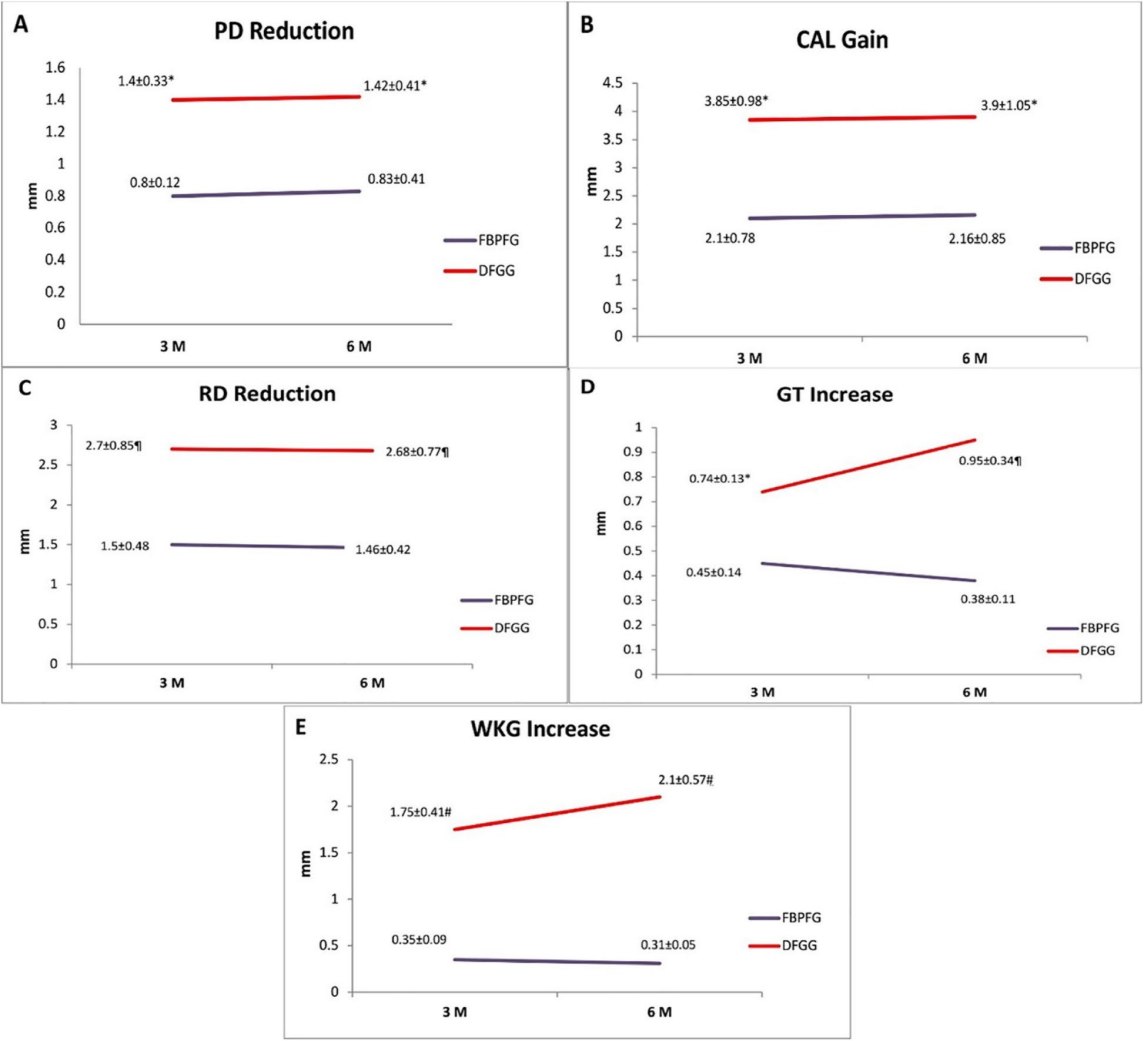
Comparison between the two studied groups at different time intervals with statistically significant differences at 3 and 6 months in **A**) PD reduction (*statistically significant difference at *P* < 0.05), **B**) CAL gain (*statistically significant difference at *P* < 0.05), **C**) RD reduction (^¶^ statistically significant difference at *P* < 0.01), **D**) GT increase (*statistically significant difference at *P* < 0.05,^¶^ statistically significant difference at *P* < 0.01), **E**) WKG increase (.^#^ statistically significant difference at *P* < 0.001)

After 3-month follow-up, group I showed a probing depth reduction (PD Reduction) of 0.8 ± 0.12 mm, while group II decreased by 1.4 ± 0.33 mm. Importantly, this difference was statistically significant (*p* < 0.05). Similarly, during the 6-month follow-up period, group I exhibited a PD reduction of 0.83 ± 0.41 mm, and group II showed a decrease of 1.42 ± 0.41 mm, with a significant statistical variation between the two groups (*p* < 0.05) (shown in Fig. 4 Panel A).

Regarding the clinical attachment level (CAL) gain, group I showed a gain of 2.10 ± 0.78 mm and 2.16 ± 0.85 mm at 3 and 6 months post-surgically, respectively, whereas group II had a CAL gain of 3.85 ± 0.98 mm and 3.9 ± 1.05 mm after 3 and 6 months; respectively. Of importance, there was a statistically significant change in group II when compared to group I at 3- and 6-month intervals (*p* < 0.05) (shown in Fig. 4 Panel B).

For reduction in recession depth (RD Reduction), which is chosen as the primary outcome of the study, group I had a decrease of 1.50 ± 0.48 mm at 3 months, while group II showed a reduction of 2.70 ± 0.85 mm, with a highly significant statistical difference concerning group I (*p* < 0.01). At 6 months, RD reduction in group I was 1.46 ± 0.42 mm, compared to 2.68 ± 0.77 mm in group II, demonstrating a highly statistically significant difference favouring group II (*p* < 0.01) (shown in Fig. 4 Panel C).

Regarding the gingival thickness increase (GT increase), at the 3-month follow-up, group I had a rise of 0.45 ± 0.14 mm. In contrast, group II showed an increase of 0.74 ± 0.13, with a significant statistical variance between the two groups (*p* < 0.05). Of interest, at the 6-month follow-up, group I showed a decrease to 0.38 ± 0.11 mm. In contrast, group II exhibited a significant increase to 0.95 ± 0.34 mm, which was highly statistically significant in comparison to group I (*p* < 0.01) (shown in Fig. 4 Panel D).

Finally, for the width of keratinized tissue (WKT), group I slightly increased by 0.35 ± 0.09 mm at 3 months. However, it slightly decreased to 0.31 ± 0.05 mm after 6 months, while group II demonstrated a more noticeable increase of 1.75 ± 0.41 mm and 2.1 ± 0.57 mm at 3 and 6 months, respectively. Most importantly, group II showed a highly significant difference at 3 and 6 months regarding WKT increase compared to group I (*p* < 0.001). Furthermore, group II revealed a statistically significant increase in WKT after 6 months compared to 3 months'values (shown in Fig. 4 Panel E).

Pain perception was evaluated using the visual analogue scale (VAS) for all participants following the surgical procedure. Table 2 shows a significant statistical change between the two groups on the first and second days, as group II experienced more nociceptive pain than group I. The two groups had no significant differences on the fourth, seventh, and fourteenth days.

**Table 2:** Visual Analog Scale (VAS) grades in the study groups at different time points

Time point	Group I (FBPFG) (*n* = 20)	Group II (DFGG) (*n* = 19)	*p*
1st day	3.70 ± 0.64^*^	4.60 ± 0.72	< *0.05*
2nd day	2.60 ± 0.47^*^	3.43 ± 0.40	< *0.05*
4th day	1.30 ± 0.25	1.60 ± 0.46	*NS*
7th day	0.28 ± 0.09	0.40 ± 0.04	*NS*
14th day	0.00 ± 0.00	0.23 ± 0.01	*NS*

^*^Statistically significant difference at *P* < *0.05* (there was statistically significant variation between the two groups on the 1 st and 2nd day of surgical intervention)

Furthermore, concerning dentinal hypersensitivity (DH), there were no significant variations between the values of both groups at baseline, 3-month, and 6-month intervals. However, there was a statistically significant change (*p* < 0.05) in both groups at the 3-month and 6-month follow-up compared to the baseline readings (as shown in Table 3).

**Table 3:** Dentinal Hypersensitivity (DH) grades in the study groups at different time points

Time interval	Group I (FBPFG) (*n* = 20)	Group II (DFGG) (*n* = 19)	*p*
Baseline	2.80 ± 0.53	2.5 ± 0.38	*NS*
After 3 months	0.43 ± 0.12^*^	0.47 ± 0.08^*^	NS
After 6 months	0.45 ± 0.14^*^	0.44 ± 0.11^*^	NS

*NS* non-significant at *P* > *0.05* (there was a non-significant difference between both groups at different time intervals)

^*^Statistically significant difference at *P* < *0.05* (statistically substantial variation within the same group at 3 and 6 months compared to baseline)

## Discussion

Gingival recession treatment modalities involve various techniques and biomaterials to accomplish aesthetic and functional needs, aiming for complete root coverage to fulfill patient desires. The coronally advanced flap (CAF) is the most frequently used procedure for root coverage trials, with the connective tissue graft (CTG) being the gold standard autogenous graft. In this RCT, the free buccal pad fat graft (FBPFG) was evaluated as an alternative autogenous graft in comparison with the de-epithelialized free gingival graft (DFGG) for localized gingival recession RT1 (Cairo et al. classification) treatment. The FBPFG is frequently applied in numerous oral surgical interventions, such as oroantral and oronasal fistula closure, and in repairs after various malignant or traumatic lesions due to its rich blood supply, adequate tissue volume, consistent quality, ability to epithelialize, safety, elasticity, and its fat-derived stem cells that promote angiogenesis and osteogenesis [17, 30]

DFGG was considered an alternative to traditional CTG harvesting methods, such as the trap door technique [31, 32]. This approach enables precise control over graft thickness and avoids deeper tissues containing fatty and glandular components, reducing patient discomfort and morbidity. Notably, the depth and height of the graft harvest are more crucial to patient outcomes than the nature of wound healing (primary or secondary) [11].

In this trial, both DFGG and BPF were utilized in conjunction with CAF, which is regarded as the most effective technique for single and multiple root coverage procedures, as it maintains blood supply to the marginal gingiva [33] and has a greater possibility of achieving complete coverage of the root surface [34]. The results indicated that the DFGG group had better outcomes in PD reduction, CAL gain, RD reduction, GT increase, and WKT increase at the 3- and 6-month follow-up compared to the BPF group.

These findings may be attributed to the effectiveness of combining CAF with DFGG, as this approach is recognized as the gold standard in GR treatment [34]. This combination enhances the likelihood of complete coverage of the root surface, gingival margin stability over time, and increases in GT and WKT due to the realignment of the mucogingival junction [34, 35]. Conversely, in the FBPFG group, achieving consistent graft dimensions proved challenging, and stabilization on the root surface was complex due to the tissue's gliding nature. Initially, there was a bulging increase in tissue thickness. Still, subsequent healing led to tissue relapse in all dimensions, resulting in incomplete root coverage, increases in PD, CAL, and RD, and decreases in GT and WKT.

Interestingly, these results contrast with those of Deliberador et al. (2015) [20], who reported comparable outcomes for subepithelial connective tissue grafts (SCTG) and non-pedicled buccal fat pad grafts, particularly noting the advantages of SCTG in long-term root coverage maintenance. They also observed an initial WKT increase after one month, consistent with our findings. They highlighted that the buccal fat pad is a structured tissue that, unlike subcutaneous fat, is not influenced by lipid metabolism, maintaining its volume for a specific duration [16]. Moreover, Kamal et al. (2021) demonstrated a significant increase in WKG in the buccal pad fat group at the 6-month follow-up after using the vestibular incision subperiosteal tunnel access technique, in comparison to the control group, which is consistent with our outcomes [36]

This RCT also revealed a significant variance in pain perception between both groups, as measured by the VAS on the 1 st and 2nd days (*p* < 0.05). In the FBPFG group, the pain was minimal compared to the DFGG group, as the graft was taken from the cheek mucosa, sutured, and allowed to heal primarily, leading to little or no pain. However, no significant statistical change was found postoperatively on the 4 th, 7 th, and 14 th days, which may be attributed to the minimally invasive DFGG harvesting technique. This technique, involving shallow depth and small size, along with gel foam dressings and crossover sutures, helped reduce patient discomfort.

Furthermore, there was no significant variance in DH between both groups. However, the two groups showed a significant statistical improvement at the 3-month and 6-month follow-ups compared to baseline (*p* < 0.05). This improvement is likely attributed to proper oral hygiene practices, thorough root surface debridement during surgery, root surface bio-modification with EDTA gel, and the successful root coverage achieved through surgical procedures.

## Conclusion and clinical significance

In conclusion, the study emphasizes that combining CAF and DFGG remains the gold standard for treating localized GR, delivering superior aesthetics and adequate keratinized tissue gain in width (WKG) and thickness (GT). This approach supports the long-term stability of the gingival margin with minimal postoperative pain and discomfort. However, FBPFG could be a suitable alternative when only a moderate reduction in GR and a modest increase in WKG and GT are required, with the added benefit of lower donor site morbidity.

## Limitations and recommendations

Due to the short-term follow-up period (6 months), the focus has primarily been on localized GR defects, and a limited number of randomized clinical trials evaluating the use of FBPFG in GR treatment, as most reported studies in the literature were case reports and case series. Therefore, we recommend conducting further multicenter randomized clinical trials that consider both localized and multiple GR defects with longer follow-up periods and larger sample sizes to more thoroughly assess the clinical outcomes of FBPFG in treating GR.

## Supplementary Information

Supplementary Material 1.

## Data Availability

The datasets used in this study are available from the corresponding author upon request.
