# Supplementary material for: The use of free buccal pad fat graft as a viable therapeutic modality in localized gingival recession: a randomized controlled clinical trial
Source: BMC Oral Health. 2025 May 24;25:780. doi: 10.1186/s12903-025-06150-8 (PMC12103013; doi:10.1186/s12903-025-06150-8)
Supplement: Supplementary file 1 — Supplementary Material 1. [file 12903_2025_6150_MOESM1_ESM.pdf]

Mansoura University
Faculty of Dentistry
Department of Oral Medicine,
Periodontology, Diagnosis, and Oral Radiology

I agree (the patient) to participate in a medical research

**Title of search**	………………………………………………………………………………………………<br>………………………………………………………………………………………………
**Steps of search**	1………………………………………………………………………………………<br>2………………………………………………………………………………………<br>3………………………………………………………………………………………<br>4………………………………………………………………………………………<br>5………………………………………………………………………………………
**Duration of search**	………………………………………………………………………………………………
**Place of Search**	………………………………………………………………………………………………
**The search results**	1………………………………………………………………………………………<br>2………………………………………………………………………………………<br>3………………………………………………………………………………………<br>4………………………………………………………………………………………
**Possible side effects**	………………………………………………………………………………………………<br>………………………………………………………………………………………………<br>………………………………………………………………………………………………

I acknowledge the undersigned that the researcher has told me that:

1 Research does not conflict with the values and ethics of society.

2 with the assurance that I kept the search secret and my right to leave it without being questioned and affecting medical care.

The name of the patient is . . . . ……. . . . .

Signed by the patient . . . ………………….. . . .

Signed by the principal investigator ……………………………………………………..
